# The Association between Admission Procalcitonin Level and The Severity of COVID-19 Pneumonia: A Retrospective Cohort Study

**DOI:** 10.3390/medicina58101389

**Published:** 2022-10-03

**Authors:** Mohamed Aon, Abdullah Alsaeedi, Azeez Alzafiri, Mohamed M. Ibrahim, Abdelrahman Al-Shammari, Omar Al-Shammari, Mahmoud Tawakul, Sherif Taha, Naser Alherz, Jarrah Alshammari, Ebraheem Albazee, Teflah Alharbi, Duaa Alshammari, Zaid Alenezi, Monerah Alenezi, Salem Aldouseri, Meshari Eyadah, Mariam Aldhafeeri, Ahmed H. Aoun

**Affiliations:** 1 Department of Internal Medicine, Faculty of Medicine, Cairo University, Giza 12613, Egypt; 2 Department of Internal Medicine, Jahra Hospital, Jahra 2675, Kuwait; 3 Jaber Al-Ahmad Military Hospital, Ministry of Defense, Kuwait City 46001, Kuwait; 4 Department of Pediatrics, Faculty of Medicine, Cairo University, Giza 12613, Egypt

**Keywords:** COVID-19, coronavirus disease, procalcitonin, mechanical ventilation, intensive care unit, mortality

## Abstract

*Background and Objectives*: An elevated procalcitonin level has classically been linked to bacterial infections. Data on the association between elevated procalcitonin and the outcome of coronavirus disease 2019 (COVID-19) are conflicting. Some linked it to associated bacterial co-infections, while others correlated the elevation with disease severity without coexisting bacterial infections. We aimed to investigate the association between high procalcitonin and the severity of COVID-19. *Materials and Methods*: Hospitalized patients with confirmed COVID-19 pneumonia were divided into two groups: the normal-procalcitonin group and the high-procalcitonin group (>0.05 ng/mL). Patients with concomitant bacterial infections on admission were excluded. The primary outcomes were the need for intensive care unit (ICU) admission, progression to invasive mechanical ventilation (IMV), and in-hospital 28-day mortality. *Results*: We included 260 patients in the normal procalcitonin group and 397 patients in the high procalcitonin group. The mean age was 55 years and 49% were females. A higher number of patients in the elevated procalcitonin group required ICU admission (32.7% vs. 16.2%, *p* < 0.001) and IMV (27.2% vs. 13.5%, *p* < 0.001). In-hospital mortality was significantly higher in the elevated procalcitonin group (18.9% vs. 8.5%, *p* < 0.001). After adjusting for other covariates, procalcitonin > 0.05 ng/mL was an independent predictor of progression to IMV (OR, 1.71; 95% CI, 1.08–2.71; *p* = 0.022), ICU admission (OR, 1.73; 95% CI, 1.13–2.66; *p* = 0.011), and in-hospital mortality (OR, 1.99; 95% CI, 1.14–3.47; *p* = 0.015). An elevated procalcitonin level was the strongest predictor of in-hospital mortality. *Conclusions*: Measurement of procalcitonin can have a prognostic role among COVID-19 patients. The admission procalcitonin level can identify patients at risk of ICU admission, progression to IMV, and in-hospital mortality.

## 1. Introduction

The coronavirus disease 2019 (COVID-19), which started as a cluster of pneumonia cases in Wuhan, China, has affected nearly all countries throughout the world [1]. Although the majority of symptomatic COVID-19 infections are mild, a subset of patients requires hospitalization due to severe COVID-19 pneumonia, and about 5% of patients develop a critical disease characterized by respiratory failure and/or other organs’ failure [2]. The early recognition of severe COVID-19 features is crucial to identify patients at risk of poor outcome, create an opportunity for early interventions, personalize the treatment protocols, allow a better allocation of valuable resources, e.g., ventilators and intensive care unit (ICU) beds, and improve the outcomes [3]. The host factors are the main determinants of the disease severity, although viral factors have also been implicated [4,5]. Clinical predictors of disease severity include demographic features such as age and sex, pre-existing comorbidities, radiographic features, and laboratory parameters [6]. Plenty of biomarkers measured in COVID-19 patients were associated with disease severity and worse outcomes such as lymphopenia and elevated D-dimer, ferritin, lactate dehydrogenase (LDH), troponin, C-reactive protein (CRP), aspartate aminotransferase (AST), alanine aminotransferase (ALT), and creatinine [7,8]. The earliest reports from China showed that elevated procalcitonin (PCT) was not a prominent feature among COVID-19 patients [9]. Subsequently, elevated PCT levels were reported more frequently in severe cases [10,11].

PCT is a glycoprotein precursor of calcitonin that does not have a hormonal action of its own. PCT level is undetectable under normal circumstances but increases with bacterial infections to adjust the host′s immune response and vasoactivity in response to bacterial sepsis [12]. Bacterial infections induce PCT production via the stimulation of macrophages which produce inflammatory cytokines such as tumor necrosis factor (TNF)-α, interleukin (IL)-1β, and IL-6 that stimulate the synthesis of PCT by all cells within few hours. On the other hand, PCT is not induced by viral infections due to TNF- α inhibition by interferon (INF)-γ [13].

Despite being a viral infection, COVID-19 may be associated with elevated levels of PCT. The elevated PCT in this setting indicates a more severe illness and a higher risk of mortality, especially among elderly patients [14]. A meta-analysis revealed that high PCT levels were associated with higher disease severity and hypothesized that this relationship suggests bacterial co-infections that increase disease severity and add to the systemic sepsis [13]. Measurement of PCT levels may identify bacterial co-infections and guide antimicrobial therapy in COVID-19 patients [15]. However, there was heterogeneity in the thresholds used and the interpretation of PCT levels [16].

On the contrary, other studies failed to find an association between PCT and bacterial co-infections in COVID-19 patients and suggested that the high PCT levels are largely related to the disease severity and the associated inflammation rather than bacterial co-infection [17,18]. Current recommendations do not support the routine use of PCT to guide decisions about antibiotics in COVID-19 and draw attention to the uncertainty about the cut-off point to be used. Institutions using PCT are advised to participate in data collection and research [19]. Further research in a variety of settings is needed to evaluate the association of PCT with disease severity, mortality, and bacterial co-infections, and the impact of PCT-guided strategies on outcomes, antibiotic prescription patterns, and safety of patients.

In response to the gaps in the literature, we aimed to investigate the hypothesis that elevated PCT is associated with increasing disease severity, need for invasive mechanical ventilation (IMV), need for ICU admission, and in-hospital mortality.

## 2. Materials and Methods

### 2.1. Study Design and Settings

Our study was a single-center retrospective cohort study. The study included patients who were admitted to the medical ward at Jahra Hospital, Kuwait (the main district hospital) with COVID-19 pneumonia between May 2020 and May 2021. Patients were divided into 2 groups according to the admission PCT level: the normal PCT group with a PCT level ≤ 0.05 ng/mL (according to the manufacturer′s reference range) and the high PCT group with a PCT level > 0.05 ng/mL. PCT was measured using an automated Enzyme-Linked Fluorescent Assay (VIDAS^®^ B.R.A.H.M.S. PCT™, BioMérieux Diagnostics, Inc., Lyon, France). All patients were treated according to the national treatment protocol that included pharmacologic thromboprophylaxis unless contraindicated and corticosteroids for hypoxemic patients (i.e., patients who need supplemental oxygen to keep SO2 ≥ 94%). Antibiotics were prescribed according to the treating physician′s discretion. Patient demographics, clinical data, and admission laboratory results were obtained from the hospital records.

### 2.2. Inclusion and Exclusion Criteria

Patients were included if they fulfilled all the following criteria: (1) admission to the medical ward with COVID-19 pneumonia (suggested by clinical and/or radiological features of lower respiratory infection); (2) COVID-19 diagnosis was confirmed with a positive reverse transcriptase-polymerase chain reaction (RT-PCR) assay for severe acute respiratory syndrome coronavirus-2 (SARS-CoV-2) on nasopharyngeal swab; (3) PCT concentration was measured on admission.

Patients who were transferred to another hospital before the outcome was known, had a bacterial co-infection, or did not have a PCT measurement on admission were excluded from the study. Bacterial co-infection was defined as positive bacterial culture from respiratory samples, blood, or urine, within 48 h from admission. Pregnant females were not included in our study.

### 2.3. Outcome Measures

The severity of COVID-19 respiratory illness on admission was assessed using a 7-category ordinal scale consisting of the following categories: (1) not hospitalized and ambulatory; (2) not hospitalized, but with limitation of activities; (3) hospitalized but did not require oxygen therapy; (4) hospitalized and required oxygen by nasal prongs or mask; (5) hospitalized and required oxygen therapy by high-flow nasal cannula or noninvasive mechanical ventilation; (6) hospitalized and required IMV; (7) hospitalized and required IMV plus additional organ support, e.g., extracorporeal membrane oxygenation, renal replacement therapy, or vasopressors [20]. The primary outcomes were the need for ICU admission, the requirement of IMV, and in-hospital 28-day mortality.

### 2.4. Statistical Analysis

Assuming an incidence of the main outcomes of 1% and 6.8% in the two groups, respectively [21], 174 patients in each group will be needed to achieve a study power of 80% and a confidence level of 95%. Validated data were tabulated, entered, and analyzed using Statistical Package for the Social Sciences (SPSS) version 22.0 (SPSS Inc., Chicago, IL, USA). Qualitative data were expressed as frequencies and percentages and comparisons between the groups were performed using the Chi-square (χ2) test. Quantitative data were expressed as mean and standard deviation (SD) if data were normally distributed. The student′s *t*-test was used for the comparison of normally distributed quantitative variables. If the quantitative data were not normally distributed, they were expressed as the median and interquartile range (IQR), and the differences between the study groups were compared by the Mann–Whitney test. Logistic regression was done to detect the independent predictors of the various primary outcomes. The association between determinants and outcome was presented as odds ratio (OR) and 95% confidence interval (CI) after controlling for other variables. A *p*-value < 0.05 was considered statistically significant.

## 3. Results

**Figure 1:**
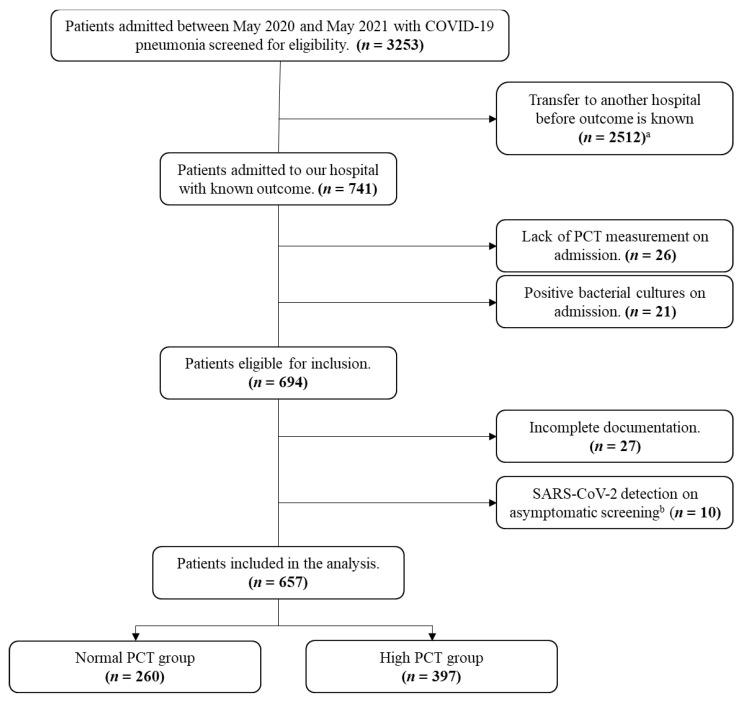
Flowchart demonstrating the patient selection process. Abbreviations: COVID-19, coronavirus disease 2019; PCT, procalcitonin; SARS-CoV-2, severe acute respiratory syndrome coronavirus-2. ^a^ Transfer of patients to a tertiary dedicated COVID-19 center, to decrease the burden on the district hospitals, was regularly arranged by the national health authorities during the pandemic. ^b^ Patients who were discovered positive on screening and referred to the medical COVID-19 ward for isolation only without evidence of pneumonia.

After assessment against inclusion and exclusion criteria, 657 patients were included in our study: 260 patients in the normal PCT group and 397 patients in the high PCT group, as shown in Figure 1.

The mean age of patients was 55 ± 15 years and 51% of them were males. Diabetes and hypertension were the most frequent comorbidities (60% and 42%, respectively). The median PCT in the normal and the high PCT groups was 0.05 and 0.21 ng/mL, respectively. Table 1 demonstrates the demographic features, comorbidities, and laboratory findings in both groups.

As demonstrated in Table 2, most patients in our cohort (89%) received empiric antibiotics therapy on admission to cover possible community-acquired pneumonia (CAP), and most of them (82%) presented with a severity scale of four. A higher number of patients deteriorated clinically in the elevated PCT group compared to the normal PCT group (*p* < 0.001), and the median length of stay was higher in the elevated PCT group compared to the normal PCT group (*p* < 0.001).

**Figure 2:**
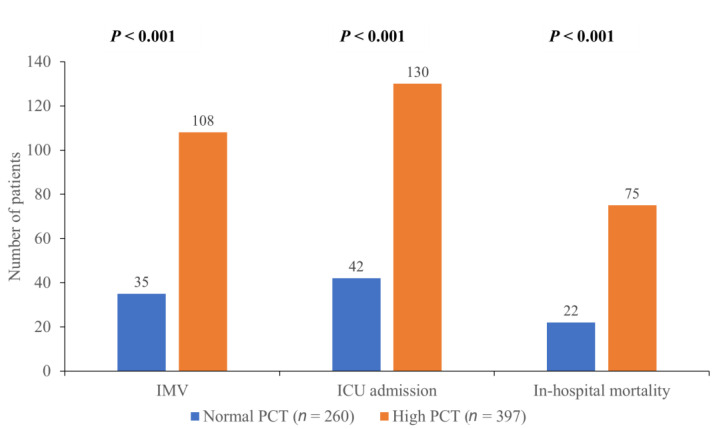
The primary outcomes in the normal and the high PCT groups. Abbreviations: ICU, intensive care unit; IMV, invasive mechanical ventilation; PCT, procalcitonin.

Figure 2 illustrates the primary outcomes in both groups. Compared to the normal PCT group, patients with elevated PCT required ICU admission and IMV more frequently (32.7% vs. 16.2% and 27.2% vs. 13.5%, respectively) and the difference between the groups was statistically significant (*p* < 0.001). The in-hospital mortality was significantly increased in the high PCT group compared to the normal PCT group (18.9% vs. 8.5%, *p* < 0.001).

### Predictors of the Primary Outcomes

**Figure 3:**
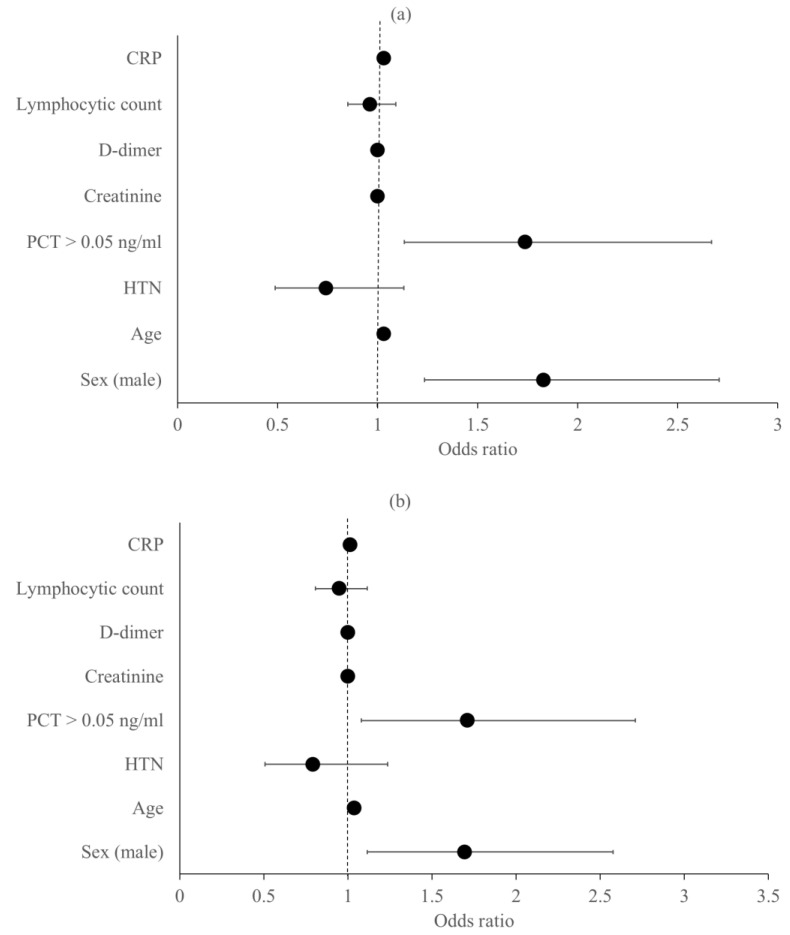

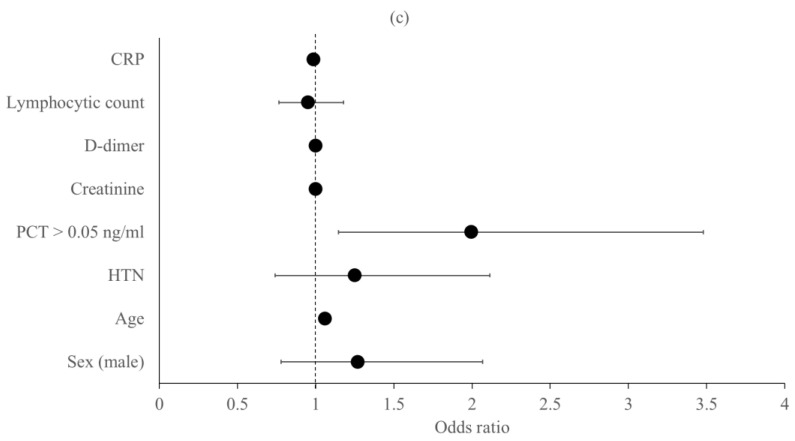
Risk factors for the main primary outcomes: (**a**) ICU admission, (**b**) IMV, and (**c**) Hospital mortality. For each variable, the black dot represents the odds ratio, and the horizontal line represents the 95% confidence interval. Abbreviations: CRP, C-reactive protein; PCT, procalcitonin; HTN, hypertension; ICU, intensive care unit; IMV, invasive mechanical ventilation.

Different models were designed, and regression analysis was performed to test the effect of the different variables on the primary outcomes (ICU admission, IMV, and in-hospital 28-day mortality). Our model identified male sex (OR, 1.82; 95% CI, 1.23–2.70; *p* = 0.003), older age (OR, 1.03; 95% CI, 1.01–1.05; *p* < 0.001), admission PCT > 0.05 ng/mL (OR, 1.73; 95% CI, 1.13–2.66; *p* = 0.011), and a higher CRP level (OR, 1.03; 95% CI, 1–1.05; *p* = 0.010) as risk factors for ICU admission after control of other variables, as demonstrated in Figure 3a. Progression to IMV predictors were male sex (OR, 1.69; 95% CI, 1.11–2.57; *p* = 0.013), older age (OR, 1.04; 95% CI, 1.02–1.05; *p* < 0.001), and admission PCT > 0.05 ng/mL (OR, 1.71; 95% CI, 1.08–2.71; *p* = 0.022) after adjusting for other covariables, as shown in Figure 3b. Older age (OR, 1.06; 95% CI, 1.04–1.08; *p* < 0.001) and admission PCT > 0.05 ng/mL (OR, 1.99; 95% CI, 1.14–3.47; *p* = 0.015) exhibited independent increasing risk of in-hospital mortality after adjusting for other covariates. As demonstrated in Figure 3c, PCT > 0.05 ng/mL was the strongest predictor of in-hospital mortality.

## 4. Discussion

In this study, we report our findings on the association between high PCT and COVID-19 severity. We found that older age and male sex were associated with disease severity; findings that have been demonstrated in previous studies [2,22]. The high PCT group patients had a higher neutrophil count and a lower lymphocytic count. These hematological parameters are known to be associated with severe COVID-19 infection [23]. Although neutrophilia is usually associated with bacterial infection, in COVID-19, it is usually linked to disease severity without clear evidence of associated bacterial co-infection. Furthermore, the use of corticosteroids contributes to the increase in the neutrophilic count. Thus, neutrophilia is not a reliable biomarker of bacterial co-infections among COVID-19 patients [18].

Other laboratory parameters, e.g., CRP, D-dimer, and creatinine were elevated in the high PCT group. These biomarkers are usually associated with more severe disease and a worse outcome [8]. Studies found that CRP levels correlate with PCT levels among COVID-19 patients [24]. Furthermore, some studies found that an elevated PCT level was associated with worsening renal functions and the development of disseminated intravascular coagulation in COVID-19 patients [25,26,27].

Consistent with previous studies, we found that increased PCT levels were associated with disease severity and the need for IMV. An early meta-analysis found that elevated PCT is associated with a 5-fold higher risk of severe COVID-19 infection [13]. Other studies found that elevated PCT levels were associated with a longer duration of IMV [18,28]. In a meta-analysis that included 3027 Chinese patients, PCT > 0.5 ng/mL was associated with progression to critical disease [29]. In another meta-analysis that included 3492 patients, Vazzana and his colleagues demonstrated that increased PCT was associated with severe COVID- 19 and a worse outcome. They suggested PCT as a specific, but not a sensitive, biomarker of COVID-19 severity [30].

SARS-CoV-2 binds to the host cells through angiotensin-converting enzyme 2 (ACE2) receptors leading to ACE2 dysregulation and renin–angiotensin–aldosterone system (RAAS) activation in several ACE2-expressing tissues, leading to a widespread inflammatory response and tissue injury [31]. The relationship between elevated PCT and COVID-19 severity can be explained by the associated systemic inflammation and the release of the proinflammatory cytokines such as IL-6, IL-1β, and TNF-α, with subsequent induction of PCT synthesis [25]. Gautam et al. demonstrated that PCT, in the setting of respiratory viral infections, may be a better indicator of disease severity than bacterial co-infections. The authors suggested that PCT induces an inflammatory response, via cytokines, which in turn increases the severity of illness and can lead to organ dysfunction and death. They also postulated that viral infections may induce PCT expression through cytokine-independent pathways [32]. In the study by Hu et al., patients with severe and critical COVID-19 had rates of bacterial co-infections that were lower than the rates of PCT elevation (20% vs. 50% and 50% vs. 80%, respectively). They suggested that, in severe and critical diseases, PCT can be a marker of disease severity independent from bacterial co-infections [33].

In our study, patients with elevated PCT had a significantly higher in-hospital 28-day mortality. In the study by Liu et al., PCT levels ≥0.05 ng/mL correlate with in-hospital mortality and worse survival [21]. Shen et al. performed a meta-analysis that included 7716 patients and showed that higher admission PCT levels were associated with disease severity and mortality [34]. 

A low burden of bacterial co-infections on admission was detected in our study (21 patients (3%)). A similar percentage of bacterial co-infections in the community-onset COVID-19 infections was detected in other studies (3.2% and 3.5%, respectively) [24,35]. A meta-analysis demonstrated that antibiotics were prescribed to 75% of COVID-19 patients, despite that the estimates of bacterial co-infections were much lesser. Of note, the Middle East region had a higher rate of antibiotic prescription (86%) compared to other regions [36]. Most of our patients received antibiotics during hospitalization. Previous studies showed that empiric antibiotics to treat CAP were administered to 91% and 77% of COVID-19 patients with high and normal PCT, respectively. Using PCT to guide antibiotic decisions in COVID-19 is biased by the possible cytokine production that may induce PCT levels [37]. The similarity of symptoms between COVID-19 and CAP has led to an upward trend of over-prescription of antibiotics [38].

The hypothesis of the association between elevated PCT and bacterial co-infections in severe COVID-19 was considered in many studies. Pink et al. demonstrated that the peak PCT levels coincide with the diagnosis of secondary bacterial infection, although it was not clear whether these infections were community-acquired or nosocomial. The authors did not establish a direct relation between high PCT levels and severity and stated that patients with secondary bacterial co-infections were expected to have worse clinical outcomes [15]. Many studies supporting PCT measurement in COVID-19 patients limit its use to antibiotics stewardship [24,39,40]. On the other hand, the guidelines endorse deficient evidence to support PCT as a marker of bacterial co-infections [19]. Fabre et al. showed that, among COVID-19 patients, admission PCT did not offer an added value to clinical evaluation in diagnosing bacterial co-infections. PCT is not uncommonly elevated in COVID-19 patients without evidence of bacterial pneumonia, thus limiting its use as a surrogate marker of bacterial infections in this setting [37]. Dolci et al. found that, among COVID-19 hospitalized patients, peak and initial PCT levels did not offer an additional value in diagnosing bacterial infections [41]. Another study from Italy demonstrated that PCT did not help distinguish COVID-19 pneumonia from non-COVID-19 pneumonia in patients presenting with respiratory symptoms and fever [42]. Vazzana et al. performed a meta-analysis that demonstrated the association between PCT and disease severity. They noticed that none of the included studies stratified PCT levels based on the presence or absence of bacterial co-infections. So, there is not enough evidence to conclude that elevated PCT in severe COVID-19 patients is related to bacterial co-infections [30]. Heer et al. found that PCT was associated with IMV requirements and inpatient death, independent of bacterial co-infections [18].

Our logistic regression model identified high PCT as a predictor of progression to IMV, ICU admission, and in-hospital mortality. In addition, PCT > 0.05 ng/mL was the strongest predictor of in-hospital mortality. Different studies have identified elevated PCT as a risk factor for worse outcomes and mortality. Xu et al. found that elevated baseline PCT is an independent predictor of mortality in hospitalized patients with COVID-19. They suggested using elevated PCT to identify patients with worse prognoses, especially among the elderly and the critically ill patients [14]. Another study found that elevated PCT, among other biomarkers, is a predictor of clinical deterioration, ICU admission, and death [8]. Sayah et al. suggested a PCT cut-off level of 0.138 and 0.16 ng/mL as predictors of severity and mortality, respectively. They found a positive correlation between the levels of IL-6 and PCT. IL-6 and PCT were the most accurate inflammatory biomarkers correlating with severity and mortality [43]. Another study found that severe and critical COVID-19 patients have higher levels of PCT and IL-6 suggesting a link between IL-6, PCT, and severity [44]. SARS-CoV-2 triggers a cytokine storm that includes a wide range of pro-inflammatory cytokines such as IL-6, IL-1β, and TNF-α. The main pathophysiological culprit of the cytokine storm in COVID-19 is IL-6. This hypercytokinemia is associated with the infiltration of lungs by inflammatory cells. Eventually, this can lead to a life-threatening multi-organ failure [45]. A study that analyzed the levels of 11 cytokines among COVID-19 patients found that the highest IL-6 levels coincide with disease severity, suggesting a strong link between this inflammatory cytokine and the pathogenesis of severe COVID-19 [4].

Although previous studies found an association between elevated PCT levels and COVID-19 severity, our study has specific features that should be emphasized. First, the timing of PCT measurement was comparable in all patients and all other laboratory parameters were measured simultaneously with PCT on admission. In addition, patients with positive bacterial cultures on admission were excluded. Finally, these findings share our real-life experience on PCT as a biomarker of COVID-19 severity. Nevertheless, our study has limitations. It was an observational retrospective study and included patients from a single hospital. Moreover, serial PCT was not measured; thus, we could not assess the dynamic PCT changes and their effect on the outcomes.

## 5. Conclusions

In conclusion, our findings demonstrate that early measurement of PCT can have a prognostic role among COVID-19 patients. Admission PCT measurement can identify patients at risk of requiring IMV and death. Our findings add to the growing evidence that elevated PCT level is a risk factor for worse COVID-19 outcomes, and we think that it can be incorporated in a practical strategy to early identify patients at risk of deterioration. Further research is required to determine the role of serial PCT measurement, the effect of drugs, and the mechanisms implicated in the association between elevated PCT and unfavorable outcomes in COVID-19. There is an urgent need to develop new and reliable strategies to diagnose bacterial co-infections among patients with COVID-19 infection.

## Figures and Tables

**Table 1:** Demographics, comorbidities, and laboratory parameters for the normal and the high PCT groups.

	Total **(*n* = 657)**	Normal PCT Group **(*n* = 260)**	High PCT Group **(*n* = 397)**	*p*-Value	NR
Age (y), mean ± SD	55.12 ± 15.1	52.55 ± 14.78	56.8 ± 15.08	<0.001	
Sex Male, *n* (%) Female, *n* (%)	335 (51) 322 (49)	107 (41.2) 153 (58.8)	228 (57.4) 169 (42.6)	<0.001	
Diabetes, *n* (%) Hypertension, *n* (%) Renal disease, *n* (%) ASCVD ^a^, *n* (%) Lung disease, *n* (%)	393 (59.8) 278 (42.3) 78 (11.9) 210 (32) 58 (8.8)	145 (55.8) 92 (35.4) 18 (6.9) 73 (28.1) 24 (9.2)	248 (62.5) 186 (46.9) 60 (15.1) 137 (34.5) 34 (8.6)	0.088 0.004 0.001 0.088 0.78	
White blood cells	6.59 (4.73–9.66)	5.6 (4.29–7.77)	7.43 (5.39–10.3)	<0.001	4–10 × 10^9^/L
Neutrophils	4.89 (3.3–7.67)	3.91 (2.7–6.14)	5.9 (3.79–8.37)	<0.001	2–7 × 10^9^/L
Lymphocytes	1.09 (0.7–1.54)	1.18 (0.8–1.7)	1 (0.65–1.48)	0.003	1–3 × 10^9^/L
Hemoglobin	128 (114–141)	128 (115–140)	128 (114–141.5)	0.807	130–170 g/L
Platelets	214 (164–265)	209.5 (159.5–263)	219 (169–265.5)	0.227	150–410 × 10^9^/L
Creatinine	83.91 (66–111.65)	75.86 (60.05–92.81)	93.24 (70.75–127)	<0.001	57–113 umol/L
ALT	30 (20–46.5)	24.5 (18–37.75)	35 (22–54.35)	<0.001	8–41 IU/L
AST	38 (28–58)	32 (25–43)	47 (30–69)	<0.001	10–40 IU/L
LDH	313 (244–388)	272 (217–320)	322 (263.5–439)	<0.001	95–200 IU/L
D-dimer	315 (205–620.8)	251.5 (168.5–433.75)	363 (240–859)	<0.001	<232 ng/ml
CRP	9.5 (6.935–13.2)	8.1 (4.93–9.65)	10.2 (7.82–15.6)	<0.001	0–0.8 mg/dl
Admission PCT	0.1 (0.05–0.265)	0.05 (0.05–0.05)	0.21 (0.11–0.56)	<0.001	≤0.05 ng/ml

^a^ Includes coronary heart disease, peripheral arterial disease, and stroke. Abbreviations: ALT, alanine aminotransferase; ASCVD, atherosclerotic cardiovascular disease; AST, aspartate aminotransferase; CRP, C-reactive protein; IQR, interquartile range; LDH, lactate dehydrogenase; NR, normal range; PCT, procalcitonin; SD, standard deviation; y, years. Note: All variables were expressed as median and IQR unless stated otherwise.

**Table 2:** The hospital course of the normal and the high PCT groups.

	Total (*n* = 657)		Normal PCT Group (*n* = 260)		High PCT Group (*n* = 397)		*p*-Value
	*n*	%	*n*	%	*n*	%	
Admission scale ^a^ 3 4 5 6	111 539 6 1	16.9 82 0.9 0.2	49 210 1 0	18.8 80.8 0.4 0	62 329 5 1	15.6 82.9 1.3 0.2	
Patients who received empiric antibiotics on admission ^b^	587	89.3	219	84.2	368	92.7	
Clinical deterioration ^c^	146	22.2	36	13.8	110	27.7	<0.001
High flow O2 therapy ^d^	226	34.4	59	22.7	167	42.1	<0.001
Hospital LOS (days) ^e^	8 (5–13)		7 (5–10)		9 (6–15)		<0.001

^a^ Details of the ordinal scale are described in the Methods section. ^b^ Antibiotics were prescribed to cover the possibility of a community-acquired pneumonia. ^c^ Defined as worsening ≥ 2 steps on the ordinal scale. ^d^ Patients who required IMV, HFNC, or NIV. ^e^ Expressed as median and IQR. Abbreviations: ICU, intensive care unit; IQR, interquartile range; HFNC, high-flow nasal cannula; LOS, length of stay; NIV, noninvasive mechanical ventilation; O2, oxygen; PCT, procalcitonin.

## Data Availability

The data supporting the findings are available from the corresponding author upon reasonable request.
